# A preliminary study about neurofilament light chain and tau protein levels in psoriasis: Correlation with disease severity

**DOI:** 10.1002/jcla.23564

**Published:** 2020-09-08

**Authors:** Gokhan Okan, Adile Merve Baki, Eda Yorulmaz, Semra Doğru‐Abbasoğlu, Pervin Vural

**Affiliations:** ^1^ Department of Dermatology Memorial Bahcelievler Hospital Istanbul Turkey; ^2^ Department of Biochemistry Istanbul Faculty of Medicine Istanbul University Istanbul Turkey; ^3^ Department of Biochemistry Memorial Bahcelievler Hospital Istanbul Turkey

**Keywords:** cognition, insulin resistance, neurofilament light chain, PASI, psoriasis, total tau protein

## Abstract

**Background:**

Studies investigating cognitive dysfunction in psoriatic patients remain inconclusive.

**Objective:**

To investigate the risk of cognitive decline in plaque‐type psoriasis patients.

**Methods:**

Serum neurofilament light chain (NFL) and tau protein concentrations in 45 patients with plaque‐type psoriasis and forty‐five healthy controls were measured by enzyme‐linked immunosorbent assay (ELISA).

**Results:**

Mean homeostasis model assessment (HOMA‐IR) values (6.82 vs 3.25) and serum levels of insulin (28.19 vs 15.71), NFL (5.74 vs 1.98), and tau (348.17 vs 207.30) in patients with psoriasis were found to be significantly higher than those of in healthy controls. There was a significant positive correlation between NFL and tau (*r* = .257, *P* = .015). There was significant correlation between NFL, tau and PASI (*r* = .310, *P* = .040) and (*r* = .383, *P* = .010), respectively. Significant correlations between NFL and insulin, TC, HDL‐C, TG, VLDL‐C, and BMI were found. NFL (9.38 vs 3.08) and tau (439.28 vs 281.58) concentrations and PASI values (23.94 vs 14.18) in patients with disease onset before 40 years were significantly higher than that of the patients with disease onset after 40 years. C‐reactive protein (CRP) was significantly correlated with BMI (*r* = .449, *P* < .001), LDL‐C (*r* = .240, *P* = .026), TG (*r* = .244, *P* = .024), and VLDL‐C (*r* = .241, *P* = .025) in patients with psoriasis.

**Conclusions:**

Increased serum NFL and tau protein levels and the presence of positive correlations between NFL, tau protein and PASI score show cognitive decline risk may be higher in moderate‐to‐severe psoriasis.

## Graphical abstract

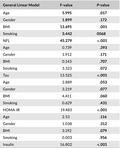

## 1 INTRODUCTION

Psoriasis is a chronic skin disease, characterized by epidermal hyperplasia, inflammation, angiogenesis, and vascular remodeling.^1^ It is caused by systemic inflammatory processes. Inflammatory myeloid dendritic cells release interleukin IL‐23 and IL‐12 that activate IL‐17 producing T cells, Th1 cells, and Th22 cells. These cells further stimulate the production of the cytokines such as IL‐1, IL‐6, and tumor necrosis factor α (TNF‐α) which have also been associated with cognitive impairment. Some studies have reported that patients with psoriasis have impaired cognitive function,^2^ an increased risk for dementia,^3^ or a genetic overlap with dementia.^4^ However, it is unclear if the association between cognitive dysfunction and psoriasis can be attributed to cardiometabolic comorbidities of psoriasis or to psoriasis per se.^5^

Neurofilaments are specifically expressed in neurons and are major cytoskeleton proteins.^6^ Their gene expression^7^ and protein phosphorylation levels^8^ directly affect axonal diameter, myelination, and conduction velocity.^9^ Upon neuronal damage, neurofilaments that are discharged into the interstitial fluid subsequently diffuse into the cerebrospinal fluid (CSF) and then the blood.^10^ Elevated levels of neurofilaments are general indicators of axonal damage in many neurological conditions, including multiple sclerosis,^11^ HIV‐associated encephalopathy,^12^ neurodegenerative disorders,^13^ aging,^14^ stroke,^15^ and traumatic brain injury.^16^ Increased levels of NFL are evident well before the clinical onset of cognitive impairments. Despite lacking diagnostic specificity, neurofilament blood levels are potentially valuable to monitor and to predict disease progression and to evaluate treatment efficacy.

Tau is microtubule‐associated protein essentially located in the axons of neurons. It is also present in glial cells to a lesser extent. It plays a fundamental role in maintaining the stability and integrity of neurons by regulating microtubule polymerization, stabilization, and suppression of microtubule dynamics.^17^ Dysregulation of the tau–microtubule complex impairs the maintenance of cellular polarity and viability.

Neurofilaments levels and tau protein are, therefore, used as surrogate markers of axonal damage. To our knowledge, this study is the first to investigate the levels of NFL and tau protein in patients with plaque‐type psoriasis, their correlation to disease severity and their relationship with other clinical and biochemical characteristics of psoriasis.

## 2 MATERIALS AND METHODS

Forty‐five patients with psoriasis were included in this study. Because this is the preliminary study, only plaque‐type psoriasis patients participated who were not previously diagnosed with psoriatic arthritis. The study lasted from February 1, 2019, to August 30, 2019. The diagnosis of psoriasis was based on clinical data.^18^ To evaluate the severity of the cutaneous manifestations of psoriasis, we used the Psoriasis Area Severity Index (PASI),^19^ which assesses erythema, infiltration, desquamation, and the percentage of the affected body surface. The PASI score ranges from 0 to 72. Higher score denotes greater severity of psoriasis. All patients were clinically assessed using PASI by the same dermatologist to ensure the same standards in establishing disease severity. Clinical and anthropometric characteristics of the patients with psoriasis are given in Table 1. The control group (collected among the hospital staff) consisted of 45 individuals matched for age and sex who underwent neurological and medical examinations to ascertain that they were free of any significant illness. None of the controls had a personal or a family history of any dermatologic disease on examination. Exclusion criteria for patients and controls were the existence of any comorbid cardiac, autoimmune, infectious, musculoskeletal, or malignant disease or a recent history of operation or trauma. Patients receiving concurrent systemic or topical anti‐psoriasis therapy were also excluded from this study. The study was approved by the Erenköy Mental and Neurological Diseases Training and Research Hospital Clinical Research Ethics Committee (07.01.2019/9‐2019). Informed consent was obtained from each subject.

**TABLE 1:** Clinical and anthropometric characteristics of patients with psoriasis

	Psoriasis
Age (y)	
Mean (range)	33.3 (17‐51)
Disease onset	
<40 y n (%)	19 (42.22)
>40 y, n (%)	26 (57.78)
Sex	
Male, n (%)	31 (68.9)
Female, n (%)	14 (31.1)
Familial history, n (%)	11 (24.4)
Smoking, n (%)	7 (15.6)
BMI, kg/m^2^ (mean, range)	26.1 (19‐38)
Systolic BP, mmHg (mean ± SD)	114.3 ± 13.3
Diastolic BP, mmHg (mean ± SD)	72.7 ± 9.2
PASI score (mean, range)	18.5 (2.0‐35.7)

Abbreviations: BMI, body mass index; BP, blood pressure; PASI, psoriasis area and severity index.

Blood samples were collected by venipuncture into gel‐separator tubes in the morning following overnight (12 hours) fasting and centrifuged within 20‐60 minutes at 2000× *g* for 10 minutes to obtain the serum samples. Routine biochemical parameters were measured on the same day, and the remaining serum in each participant's sample was stored at −80º C for further biochemical analysis of the markers specific to this study. Serum glucose, total cholesterol (TC), triglyceride (TG), high‐density lipoprotein cholesterol (HDL‐C), C‐reactive protein (CRP), insulin, and low‐density lipoprotein cholesterol (LDL‐C, when TG was above 350 mg/dL) were determined by using commercially available assay kits with autoanalyzer (Architect CI 8200; Abbott Diagnostics). In subjects with TG levels below 350 mg/dL, low‐density lipoprotein cholesterol (LDL‐C) levels were calculated by Friedewald formula: LDL‐C = TC (mg/dL) − [(TG/5 (mg/dL) + HDL‐C (mg/dL)].^20^ VLDL‐C was calculated as TG/5. Homeostatic model assessment of insulin resistance (HOMA‐IR) was computed using the following formula: HOMA‐IR = [fasting insulin (µU/mL) × fasting glucose (mg/dL)]/405)].^21^

Total levels of tau protein (Cat.No E1333Hu) and NFL (Cat.No E446Hu) were assayed using commercially available enzyme‐linked immunosorbent assay (ELISA) kits (Bioassay Technology Laboratory). The sensitivity of this ELISA kit was 12.31 ng/L for tau protein and 0.054 ng/mL for NFL. The intra‐assay and inter‐assay coefficients of variation for the two parameters were <8% and <10%, respectively.

### 2.1 Statistical analysis

All statistical analyses were performed using IBM SPSS statistical analysis software for Windows (version 21; SPSS Inc). Mann‐Whitney *U* and Spearman correlation tests were used for the evaluation of clinical and biochemical parameters. Univariate analysis of variance (GLM, Univariate; IBM) was used to construct the model for explaining the variations observed in NFL, tau, HOMA‐IR, and insulin. Age, gender, body mass index (BMI), and smoking status were included as covariates.

## 3 RESULTS

NFL and tau were analyzed in 45 patients with psoriasis (14 women and 31 men) and in 45 age‐matched healthy controls (16 women and 29 men). The mean age (range) and BMI (range) of the controls were 33.3 (17‐56) years and 26.9 (18‐37) kg/m^2^, respectively. The values of the systolic and diastolic blood pressure (BP) (mean ± SD) of the controls were 111.2 ± 11.5 mmHg and 75.7 ± 7.5 mmHg, respectively. Eight controls (17.8%) smoked. Lipid profile and insulin resistance parameters recorded in controls and patients with psoriasis are given in Table 2. There were no significant differences between the groups in terms of age, gender, BMI, systolic‐diastolic BP values, and lipid profile parameters. Mean insulin levels and HOMA‐IR values were significantly increased in patients compared with those in controls (*P* = .001 and *P* < .001 respectively). In addition, serum NFL and tau levels in the patients with psoriasis were increased (*P* < .001 and *P* = .011, respectively) compared with those in healthy controls (Table 2).

**TABLE 2:** Lipid profile, insulin resistance parameters, and serum NFL and total tau protein (tau) in healthy controls and patients with psoriasis (mean, range)

	Control	Psoriasis	*P‐*value
TC, mg/dL	155.11 (86‐329)	165.88 (51‐245)	.113
TG, mg/dL	97.27 (28‐402)	119.18 (12‐389)	.119
HDL‐C, mg/dL	37.20 (15‐60)	42.90 (20‐76)	.068
LDL‐C, mg/dL	98.93 (16‐204)	98.93 (22‐162)	.470
VLDL‐C, mg/dL	19.47 (6‐80)	23.81 (2‐78)	.121
Glucose, mg/dL	86.84 (60‐166)	92.45 (50‐144)	.227
Insulin, µIU/ml	15.71 (4.30‐48.8)	28.19 (1.8‐77.9)	.001
HOMA‐IR	3.25 (0.69‐11.85)	6.82 (1.17‐27.70)	<.001
CRP, mg/L	3.01 (0.20‐11.40)	2.17 (0.40‐7.30)	NS
NFL, ng/mL	1.98 (0.42‐7.45)	5.74 (0.64‐15.78)	<.001
Tau, ng/L	207.30 (91.83‐462.32)	348.17 (82.52‐978.10)	.011

Note

Mann‐Whitney *U* test, data are presented as median (range).

Abbreviations: CRP, C‐reactive protein; HDL‐C, high‐density lipoprotein cholesterol; HOMA‐IR, homeostasis model assessment; LDL‐C, low‐density lipoprotein cholesterol; NFL, neurofilament light chain; PASI, psoriasis area and severity index; TC, total cholesterol; TG, triglyceride; VLDL‐C, very‐low‐density lipoprotein cholesterol.

We further performed analysis of covariance to investigate the influence of factors such as age, gender, BMI, and smoking status on NFL, tau, HOMA‐IR, and insulin. The levels of NFL, tau, and insulin and the HOMA‐IR values remained significantly increased in the patients with psoriasis compared to those in controls upon stratification by age, gender, BMI, and smoking status (Table 3).

**TABLE 3:** General linear model for NFL, total tau protein (tau), HOMA‐IR, and insulin significance taking age, gender, BMI, and smoking status as covariates

General Lineal Model	F*‐*value	*P*‐value
Age	5.995	.017
Gender	1.899	.172
BMI	13.695	.001
Smoking	3.442	0068
NFL	45.279	<.001
Age	0.739	.393
Gender	1.912	.171
BMI	0.143	.707
Smoking	3.323	.072
Tau	13.525	<.001
Age	3.889	.053
Gender	3.219	.077
BMI	4.411	.060
Smoking	0.629	.431
HOMA‐IR	19.483	<.001
Age	2.53	.116
Gender	1.038	.312
BMI	3.192	.079
Smoking	0.003	.956
Insulin	16.802	<.001

Abbreviations: BMI, body mass index; HOMA‐IR, homeostasis model assessment; NFL, neurofilament light chain.

There was a significant positive correlation between NFL and tau (*r* = .257, *P* = .015). Also, significant correlations between NFL, tau and PASI (*r* = .310, *P* = .040) and (*r* = .383, *P* = .010), respectively, were found. In addition, NFL was significantly correlated with insulin, TC, HDL‐C, TG, and VLDL‐C levels and BMI (Table 4). Additionally, CRP correlated significantly with LDL‐C, TG and VLDL‐C levels, and BMI (Table 4). As we expected, there were strong correlations between parameters of insulin resistance and lipid profile (data not shown).

**TABLE 4:** Correlations between NFL, CRP, and some study parameters

	NFL *r*, *P*‐value	CRP *r*, *P*‐value
Insulin	.240 (.023)	.038 (.725)
TC	.273 (.011)	.196 (.071)
HDL‐C	.218 (.042)	−.210 (.052)
TG	.213 (.047)	.244 (.024)
LDL‐C	.203 (.060)	.240 (.026)
VLDL‐C	.211 (.050)	.241 (.025)
BMI	.238 (.031)	.449 (<.001)

Abbreviations: BMI, body mass index; CRP, C‐reactive protein; HDL‐C, high‐density lipoprotein cholesterol; LDL‐C, low‐density lipoprotein cholesterol; NFL, neurofilament light chain; TC, total cholesterol; TG, triglyceride; VLDL‐C, very‐low‐density lipoprotein cholesterol.

In addition, we evaluated the effect of age of disease onset of psoriasis on NFL, tau, and PASI (Table 5). NFL and tau concentrations and PASI score in patients with disease onset before 40 years were significantly higher than those observed in patients with disease onset after 40 years.

**TABLE 5:** Serum neurofilament light chain (NFL), total tau protein (tau) concentrations, and psoriasis area and severity index (PASI) in patients with psoriasis with disease onset before 40 y, and after 40 y (mean, range)

	Before 40 y	After 40 y	*P‐*value
NFL, ng/mL	9.36 (1.42‐15.78)	3.08 (0.64‐7.00)	<.001
Tau, ng/L	439.28 (135.09‐978.10)	281.58 (82.52‐767.87)	.05
PASI	23.94 (2.50‐35.7)	14.18 (2.00‐35.7)	.003

Note

Mann‐Whitney *U* test.

Abbreviations: NFL, Neurofilament Light Chain; PASI, Psoriasis Area and Severity Index.

## 4 DISCUSSION

In this study, we investigated the correlation of the levels of serum NFL and tau protein with PASI scores. The results of present study demonstrated that (a) increased insulin and HOMA‐IR values in patients indicated the presence of insulin resistance in psoriasis; (b) NFL and tau protein levels were increased in patients compared with those in controls; (c) NFL and tau protein levels were significantly correlated with PASI score; (d) levels of NFL were significantly correlated with insulin, TC, HDL‐C, TG and VLDL‐C levels, and BMI; (e) there were significant correlations between CRP and LDL‐C, TG and VLDL‐C, and BMI; (f) NFL and tau concentrations and PASI score in patients with disease onset before 40 years were significantly higher than those observed in patients with disease onset after 40 years. These findings suggest that the risk of mild cognitive impairment may be higher in moderate‐to‐severe psoriasis.

The pathogenesis of dementia remains elusive. However, the presence of amyloid deposition, vascular injury, and inflammation have been implicated to contribute to the pathogenesis of dementia.^22^ Mild cognitive impairment (MCI)—wherein objective cognitive impairment does not affect daily living activities—is considered a transitional stage between normal aging and dementia. In some cases, MCI does not worsen and the affected individuals may even revert back to cognitive normality. Morphological abnormalities in the brain that characterize dementia may appear in people diagnosed with MCI 20 or more years before signs of the disease develop.^23^ Therefore, most studies are prone to underestimated the risk for dementia because of limited long‐term follow‐up. Epidemiological studies have shown that diabetes mellitus and insulin resistance are strong risk factors for cognitive decline and dementia^24^ Impaired glucose metabolism has also been shown to be associated with increased progression from mild cognitive impairment to dementia. Hypertension, dyslipidemia, metabolic syndrome, and midlife obesity are other risk factors. Psoriasis has also been found to be associated with an increased risk of cognitive impairment and dementia. Two main proposed explanations for an association between psoriasis and cognition are shared genetic polymorphisms and a common inflammatory pathway. Firstly, gene polymorphisms of apolipoprotein E, which plays a role in modulating inflammation, have been associated with both psoriasis and dementia.^25^ Secondly, the two diseases also share inflammatory pathogenesis involving TNF, interleukin‐17, and interleukin‐23. Systemic inflammation has been found to be associated with increased microglia activation and cerebral inflammation providing a key mechanistic link between psoriasis and impaired cognition.^2^

Neurofilaments are intracellular filaments found in the central and peripheral nervous systems. They are composed of four subunits: neurofilament light chain, neurofilament medium chain, neurofilament heavy chain, and alpha internexin. Neurofilament light chain is the smallest of the neurofilament triplet proteins that are structural component of axons. NFL release is accelerated in response to axonal damage, which can be caused by neurodegeneration, inflammation, trauma, or stroke. Disruption of axons or death of neurons releases NFLs into the CSF and resulting in its extensive distribution in both the CSF and blood. On the other hand, tau protein is a microtubule‐associated protein mainly located in the neuronal axons. It is essential in maintaining the stability and integrity of neurons by regulating the microtubule. Neurofilaments and tau in the brain can reach the periphery through disrupted blood–brain barrier and receptor mediated mechanisms. Increased blood NFL and tau protein concentrations were reported in neurodegenerative and neuroinflammatory disorders. Since tau pathology is more strongly associated with clinical and cognitive decline than amyloid pathology, and tau may accumulate in susceptible regions earlier than amyloid, we measured NFL and tau proteins in the blood.^26^ Several studies have shown increased plasma tau in individuals with Alzheimer's disease (AD) compared with that in healthy individuals. In a study of normal elderly people without cognitive impairment, elevated levels of NFL in CSF were observed in those who developed cognitive decline during follow‐up.^27^ The measurement of NFL in blood could be useful for preventative screening of preclinical stages of AD. Framingham Dementia Study indicated that higher plasma tau concentrations were associated with a higher risk of AD, suggesting that plasma tau might help predict dementia even in individuals who are cognitively unimpaired.^28^ In a similar report from Mayo Clinic Study of Aging, a significant reduction in global cognition, memory, attention, and visuospatial ability was predicted by high plasma tau concentrations in individuals with MCI and no cognitive impairment.^29^

Psoriasis has been found to be associated with an increased risk of cognitive impairment and dementia in most studies.^30^ Marek‐Jozefowicz et al performed a cross‐sectional study to evaluate cognitive function in 97 severe psoriasis cases and 91 controls. Psoriasis cases scored significantly lower on cognitive tests than controls, especially with regard to working memory. While severity of psoriasis was not associated with cognitive function, longer duration of disease dramatically correlated with worse cognitive test results.^31^

Pezzolo et al identified 318 psoriasis cases (moderate‐to‐severe cases accounted for 23.2%) for comparison with 9678 controls. The prevalence of MCI in patients with psoriasis was not significantly higher than in patients without psoriasis. It was the only study which reported a reduced risk for both MCI and dementia in psoriasis patients compared with that in controls. Pezzolo et al proposed that the increased use of hypertensive medication and methotrexate in psoriasis group could be possibly protective against dementia. In their study, the psoriasis patients included those with milder disease which may explain the lower risk of cognitive dysfunction. Subgroup analysis based on psoriasis severity was not performed by Pezzolo et al because of the low number of dementia cases.^32^

Colgecen et al compared 77 psoriasis cases with 83 controls. Psoriasis patients performed dramatically poor in the visuospatial and executive functioning domains compared to the controls. However, no correlation was found between the severity of or duration of psoriasis and cognitive dysfunction. The study primarily included patients with higher disease severity or those undergoing systemic medical treatment; therefore, a true comparison patients with mild psoriasis was not performed.^33^

Epidemiological evidence suggests that type 2 diabetes mellitus and insulin resistance are risk factors for developing AD and increase the likelihood of developing late‐onset AD.^34^ Obesity may also accelerate the progression of cognitive decline and dementia. Tau pathologies were observed in type 2 diabetes mellitus patients.^35^ Since psoriasis shares many biochemical features of insulin resistance and metabolic syndrome, the elevation of tau protein and neurofilament in psoriatic patients is not surprising. Moreover, our results showed that tau protein and NFL levels were significantly correlated with PASI score, suggesting that NFL together with tau protein may be a marker for psoriasis severity. The difference observed between our study and by the Pezzalo group could be explained by the difference in severity of psoriasis in the participants: psoriasis was predominantly milder in their study participants, and this may explain the lower risk of cognitive dysfunction observed in their study.

Studies in older adults rather than middle aged adults suggest that a higher BMI may be associated with a lower risk of cognitive decline, dementia, and AD.^36^, ^37^ Obesity may influence long‐term risk of AD but the early or preclinical stages of AD may be associated with weight loss. In our study, it may explain higher NFL and tau concentrations and PASI score in patients with disease onset before 40 years than that of patients with disease onset after 40 years.

Our study has some limitations. First, we had a small number of participants. A larger population is needed to support the generalization of these results. Second, the current study did not perform amlyloid‐beta positron emission tomography or provide neuropathological confirmation of dementia. Therefore, further studies with a larger number of patients and long enough follow‐up periods will help us better understand the relationship between psoriasis and impaired cognition.

## 5 CONCLUSIONS

Psoriasis is multisystem inflammatory disease associated with numerous comorbidities. Psoriasis patients might have early and subtle cognitive impairment or may develop it during the course of the disease. Patients with psoriasis may need through neurological evaluation in order to detect early MCI. Systemic therapy for psoriasis or biologicals used earlier in the treatment protocol may change the natural history of disease and might improve cognitive impairment. More research will be necessary to refine and further elaborate our findings.

## CONFLICT OF INTEREST

The authors declare no conflicts of interest.

## AUTHOR'S CONTRIBUTION

All authors read and approved the final version of the manuscript.
